# Self-monitoring of blood glucose in Black Caribbean and South Asian Canadians with non-insulin treated Type 2 diabetes mellitus: a qualitative study of patients’ perspectives

**DOI:** 10.1186/1472-6823-13-46

**Published:** 2013-10-14

**Authors:** Enza Gucciardi, Mariella Fortugno, Andrea Senchuk, Heather Beanlands, Elizabeth McCay, Elizabeth E Peel

**Affiliations:** 1 School of Nutrition, Ryerson University, 350 Victoria Street, Toronto, Ontario, M5B 2K3, Canada; 2 Daphne Cockwell School of Nursing, Ryerson University, Toronto, Ontario, Canada; 3 School of Life & Health Sciences, Aston University, Birmingham, UK

**Keywords:** Self-monitoring of blood glucose, Diabetes mellitus, Caribbean, South Asian

## Abstract

**Background:**

To examine the views and current practice of SMBG among Black Caribbean and South Asian individuals with non-insulin treated Type 2 diabetes mellitus.

**Methods:**

Twelve participants completed semi-structured interviews that were guided by the Health Belief Model and analyzed using thematic network analysis.

**Results:**

The frequency of monitoring among participants varied from several times a day to once per week. Most participants expressed similar experiences regarding their views and practices of SMBG. Minor differences across gender and culture were observed. All participants understood the benefits, but not all viewed SMBG as beneficial to their personal diabetes management. SMBG can facilitate a better understanding and maintenance of self-care behaviours. However, it can trigger both positive and negative emotional responses, such as a sense of disappointment when high readings are not anticipated, resulting in emotional distress. Health care professionals play a key role in the way SMBG is perceived and used by patients.

**Conclusion:**

While the majority of participants value SMBG as a self-management tool, barriers exist that impede its practice, particularly its cost. How individuals cope with these barriers is integral to understanding why some patients adopt SMBG more than others.

## Background

Self-monitoring of blood glucose (SMBG) is currently recommended as a tool for the self-management of Type 1 and Type 2 diabetes mellitus (DM) [1]. SMBG provides patients with information on the effect that self-management behaviours (e.g., diet, exercise, medication) have on blood sugar levels, which may promote better management of these behaviours and improve glycemic control. However, research on the effectiveness of SMBG for non-insulin-treated patients is inconclusive and highly debatable. Some studies have found that SMBG does not improve glycemic control [2,3], while systematic literature reviews [4-7] and meta-analyses [8-11] report a modest improvement in glycemic control (reduction of HbA1c from 0.21 to 0.38%) in this population. The clinical significance of these modest improvements are questionable, as demonstrated by a recent study utilizing the United Kingdom Prospective Diabetes Study Outcomes Model to forecast diabetes-related complications, quality-adjusted life years and costs over a 40 year period [12]. Findings indicate that the modest mean reduction of HbA1c of 0.25% [7] predicts an absolute risk reduction of diabetes complications by less than 1% for patients who monitor for 40 years [12]. As a result, it has been argued that SMBG not be performed routinely by this population [13]. Proponents of SMBG have suggested that monitoring should be evaluated as part of a multifaceted self-management program, rather than as a sole self-management intervention [14,15].

Debate on the cost-effectiveness of SMBG also continues among researchers, healthcare professionals and policy-makers because SMBG requires patients to purchase blood glucose strips, lancets, and glucometers [16,17]. In Canada, reimbursement of these costs varies by province, ranging from full coverage to none [18]. Despite the uncertainty of SMBG’s clinical and cost-effectiveness, it is important to assess patients’ views on the utility of this practice, as they are primarily responsible for their management. In fact, patients’ perspective on SMBG practice was identified as a main knowledge gap at a conference on SMBG held in Canada in 2006 [19]. Only two qualitative British studies [20,21], following a white sample for 4 years, explored SMBG among non-insulin-treated patients. These studies found that although SMBG can increase patients’ awareness of how self-management behaviours affect blood sugar levels, it can also trigger feelings of anxiety, guilt, and self-blame. In addition, health care professionals’ interest in patients’ readings had an important impact on whether patients practice SMBG.

Given that the only research available regarding the experiential views and practice of SMBG was conducted in a white population, more research is needed on other ethnic populations that share a greater burden of the diabetes prevalence [22]. For instance, people of South Asian or Black Caribbean ancestry [23,24], including recent immigrants [25], have a disproportionately higher prevalence of type 2 DM in Canada and worldwide; however, they have not been well represented in the diabetes literature. Patients’ experiences, thoughts and opinions about SMBG need to be accounted for and considered in order to suitably inform clinical practice and public policy in the treatment and management of patients with non-insulin treated diabetes. The objective of our study was to explore the perceived value and practice of SMBG among South Asian and Black Caribbean individuals with non-insulin-treated type 2 diabetes using the Health Belief Model [26]. Findings from this study will contribute to the discussion on the utility of SMBG by giving voice to populations that are disproportionately affected by diabetes.

## Methods

### Recruitment and sample

Participants were primarily recruited from community health centres and diabetes education centres in the Greater Toronto Area, in Ontario, Canada. Following ethics approval by all recruitment sites, health care providers were asked to promote the study and provide contact information for patients who were interested in participating. Participants were eligible if they had Type 2 DM and were not using insulin, were over 18 years old, spoke English, had a history of monitoring their blood sugar levels, and self-identified as being of Black Caribbean or South Asian ethnicity. An honorarium was offered to cover travel and parking costs. Through purposive and snowball sampling, 12 participants were recruited (six from each cultural group) with an equal number of men and women. Further demographic information is provided in Table 1.

**Table 1:** Demographic details of participants (n = 12)

	**Number of participants (n)**		
	**Total sample**^**1**^	**Black Caribbean**	**South Asian**
**Mean age**	56.5 (n = 10)	58 (n = 5)	55 (n = 5)
**Mean years since diagnosis**	6.1 (12)	7.5 (6)	4.5 (6)
**Mean years living in Canada**	19 (9)	32 (4)	12 (5)
**Marital status (n)**			
Married	8	2	6
Single	2	2	0
**Employment (n)**			
Employed	4	1	3
Part-time	2	0	2
Unemployed and looking	3	2	1
Retired	1	1	0
**Education (n)**			
< Grade 9	1	0	1
Some High School	1	1	0
High School	2	1	1
Some post-secondary	1	1	0
Post-secondary	4	1	3
**Frequency of SMBG (n)**			
≥ 1 time/day	7	5	2
2-4 times/week	4	1	3
1 time/week	1	0	1

^1^Total sample does not add up to 12 due to missing data.

### Data collection

Semi-structured interviews were conducted in 2009 and 2010 in English by two research assistants trained in qualitative interviewing at a location convenient for the participant. Interviews were approximately 45 minutes in length, were audio-recorded with participants’ consent, and transcribed verbatim. Participants were asked to complete a demographic questionnaire prior to the interview; however, a few participants did not fully complete it. Participants are identified by ethnicity, gender and number (e.g., CW1 refers to Participant 1, who is a Caribbean women; SAM2 refers to Participant 2, who is a South Asian man).

The interview guide (see next paragraph) that was developed by the research team, and subsequent data analyses were based on the Health Belief Model (HBM), a socio-psychological model that attempts to explain and predict health behaviours [26]. The HBM assumes that a person will take a health-related action if they: 1) feel that a negative health condition can be avoided; 2) expect that they can avoid a negative condition with a recommended action; and 3) believe that they can successfully perform the recommended action. The HBM has six major components: (a) people perceive themselves at risk of a health condition or complication (perceived susceptibility); (b) people perceive that the health condition or complication is serious (perceived severity); (c) people believe that they can avoid a negative condition with a recommended action (perceived benefits); (d) there are few self-identified psychosocial costs and barriers to performing the behavior (perceived barriers); (e) consistent stimuli within the environment promote the recommended behaviour (cues to action); and (f) people have confidence in their ability to successfully perform the recommended action (self-efficacy) [26]. The HBM has been successfully used in previous diabetes research exploring health beliefs [27] among various ethnic groups [28,29]. Given that SMBG is a health-related action that can potentially help people manage their diabetes to avoid the risk of, or delay, further health complications, the HBM is an appropriate conceptual model to explore the perceived value and practice of SMBG.

### Semi-structured interview guide

*Background Information/Warm-up*

•Let me start off by asking you a few question about yourself. How long have you had diabetes?

•How do you manage your diabetes?

*Perceived Severity/Susceptibility*

•How serious do you think diabetes is?

•Describe how diabetes has affected you current health.

•Describe how you think diabetes will affect your health in the future.

•Describe what you think are your chances of developing a diabetes complication. Why you think that?

•How do you think monitoring your blood sugar can affect your future health and chances of developing complications?

*Perceived Benefits of Self-monitoring*

•How often do you self-monitor your blood sugar levels?

•Tell me about your experiences with testing your blood sugar levels.

•What do you think are the benefits of testing?

•Describe how testing your blood sugar either helps or does not help you manage your diabetes.

•Describe how testing your blood sugar influences you to perform or not perform other management activities (such as diet, medication taking, and exercise?)

*Perceived Barriers*

•Describe any difficulties you have when testing your blood sugar.

**Probes:** (Stress caused by SMBG? Emotions experienced after SMBG? How do you feel about pricking your skin?

•Some people do not test their blood sugar regularly. What do you think are reasons why people do not test regularly?

•Describe what can prevent you from testing your blood sugars regularly (What kept you from testing?)

**Probes:** (Any aspect of your life? Perceived barriers? Financial reasons? Lack of family support?)

*Cues to Action*

•When you last saw your doctor, what advice were you given about testing your blood sugar at home?

•Describe your conversations or the types of questions your doctor asks you about your blood sugar levels and your readings at each visit.

**Probes:** (Does s/he answer your questions? Is s/he clear when talking to you about testing?)

•Describe how important you think it is to your doctor that you test your blood sugar levels at home.

•How does your doctor affect your testing of your blood sugar?

•If you thought that testing blood sugars was not important to your doctor, how would this affect your testing of blood sugar?

*Self-efficacy (Knowledge, Capability and Confidence)*

•Describe what a high and low blood sugar reading means to you.

**Probes:** (Examples of readings you have had? What did you think when you got your reading?)

•Describe any actions you take to control your blood sugar levels when you get high and low readings.

**Probes:** (Any lifestyle changes or behaviours?)

•Describe how confident you are in your ability to monitor your blood sugar levels.

**Probes:** (Using your equipment at home?)

•How confident are you about your ability to control you blood sugar levels within recommended the range?

•Describe how testing your blood sugar affects your ability to manage your diabetes.

**Probes:** (Does it make it easier? Harder? How so?)

•How confident are you in operating the glucometer?

•How comfortable are you with your level of knowledge about monitoring your blood sugars? Why do you feel that way?

•Describe any experiences you have had, if any, when you were unsure about why you were getting certain blood sugar readings.

*Other Variables*

•Please describe how testing your blood sugar may affect other parts of your daily and family life.

*Cool-Down/Wrap-up Questions*

•Is there anything else I haven’t asked you about that you would like to add?

•The responses you have provided may lead to some more questions. If so, can we contact you for a brief telephone interview?

### Data analysis

Interview transcripts were kept in password protected and secure hard drives at our research lab throughout all stages of data analysis to ensure confidentiality and data security. Following initial data collection, all identifying information of the participants was removed and objective identifiers were used on the transcripts to ensure anonymity. Interview transcripts were imported into NVivo 8, where thematic networks analysis [30] was used to analyze the interview data. We selected this study technique because there is limited research that has focused on narrative accounts of how men and women perceive and value the use of SMBG in their diabetes self-management.

Our research team was composed of two academic experts in the area of diabetes (EG & EP), two research assistants training in the area of nutrition (MF & AS), one academic expert in psychosocial issues in chronic disease (HB), and one academic expert in mental health and self-concept of illness (EM). To facilitate dependability (analogous to reliability in quantitative research), three authors (EG, MF, AS) open coded transcripts independently, narrowed data into more concise ideas, identified salient quotes or recurrent phrases and discussed their analyses at regular meetings until they reached consensus. Emerging themes were organized using the HBM; thus, deductive data analysis was conducted. Any thematic patterns specific to gender, within and across cultural groups, as well as participants who reported divergent experiences were noted and discussed at research team meetings. Ethics approval for this research study was obtained from Ryerson University Research Ethics Board.

## Results

All participants reported that their physician had recommended that they use SMBG, and all acknowledged SMBG as an important tool in the general management of diabetes. All participants had access to at least one glucometer, and all stated they were practicing SMBG at least once a week. Most participants had been given a glucometer and instructed on its use by their physician or a diabetes educator. The frequency of SMBG varied among participants, from several times a day to once a week (see Table 1). Most Caribbean participants practiced SMBG once a day or more, whereas most South Asian participants practiced it once a day or less. Some participants kept log books provided by their physician or diabetes educator, in which they recorded their blood glucose results. Although there was variation across participants in years since diagnosis of diabetes, diabetes symptoms, level of diabetes education, and treatment plan, their experiences practicing SMBG were somewhat similar. In the following sections, emerging themes representing participants’ perspectives on SMBG are organized according to the HBM and are presented with participant quotes.

### Theme one: perceived severity of diabetes and susceptibility of future complications

All participants expressed the belief that their chances of future diabetes-related complications are high, given the severity of the disease. Recognizing that diabetes is manageable yet progressive, most participants perceived themselves to be susceptible to complications, and all expressed uncertainty about their future health. These findings were evident in the fear that several participants described regarding further complications (i.e., losing their sight or a limb) and of possibly needing insulin injections in the future.

**CW3:** That I’m gonna go blind, or lose one of my…that I’ll still be able to get around. My nurse told me that the next time I go to my doctor, ask her to send me to an eye specialist to examine my eyes because with the diabetes, it might be bleeding at the back of the eyes or something so, that is scary. Especially to think that you might lose your sight, that definitely is scary. It’s even more scary than losing a limb, because if I lose a foot I can still get around. So that is scary.

However, one woman participant who had been diagnosed with diabetes a year before the interview, had yet to experience symptoms. Being both newly diagnosed and asymptomatic diminished her perception of susceptibility to future complications despite her awareness of the severity of her illness and familiarity with others suffering from diabetes related complications. This participant was also the least likely to practice SMBG (i.e., once a week) among all participants.

**SAW7**: …and I don’t take it [diabetes] seriously. That’s because, as I told you, I don’t have any physical symptoms. There was nothing much. I’m only at the border line. Nothing’s going to happen to me. I know it’s not true, but I keep telling myself that is not, that nothing will happen to me. Things happen to everybody else, but things are not going to affect me. So it’s a false world we live in. I know I should be taking it seriously.

### Theme two: perceived benefits of SMBG

When participants were asked about the benefits of SMBG, they all acknowledged that it is a valuable tool for the general management of diabetes, regardless of how often they practiced. Most used SMBG to gauge and maintain other self-management activities, such as diet, physical activity and taking medications that affect their blood glucose levels.

**SAM12**: When you see your blood sugar is within normal range, then you don’t feel anything. If it is going up then you have to start thinking, what is wrong – the type of food I’m eating yesterday or the day before – that it is not up to the mark, or I’ve taken some more type of carbohydrate. So then even the knowledge – be careful, it gives you the signal. When you check your blood sugar, you can see the medicine that you are taking is of right doses, the food that you’re taking is also the correct categories. Exercise, oh if I miss my exercise for a day or two, it [glucose level] remains up to 7, 7.5. The day I do my exercise for about say 30 minutes, 20 minutes, the next morning the blood sugar is 6.5, 6.2.

A few men expressed a strong desire to practice SMBG as a way to understand what was physiologically happening within their bodies. As one participant (CM4) described, “If normally, I’m going out, I usually take it [SMBG] to know where I stand, y’understand, it’s very good, like, you’re gonna be out maybe for the day, it’s very good to take it to know where you stand.” An asymptomatic, newly diagnosed man (SAM6) conveyed similar sentiments: “It [SMBG] is important, you know. I just want to know it, I just want to know it, what is the level of my blood sugar. That’s the only thing because, I don’t feel [any symptoms] anything you know.

Other participants considered SMBG to be essential in their daily management. For instance, one participant (CM8) used the phrases “lifeline”, “you’re your own doctor”, and “it’s like a medication, I have to bring [it everywhere]” when describing the need to perform SMBG. Furthermore, blood glucose results within recommended range were described as encouraging and reinforcing self-management:

SAM12: When I see the reading is below 7, what the doctor advised me, I feel happy. I think mentally, I’m not worried about it. I know it [diabetes management] is improving.

SAW11: Like, monitoring helps you. Like if it’s fine, you can…control more. Monitoring is a check and balance. It gives you a check. It encourages… It does encourage me. It lets you know what it [glucose level] is and, if it’s high.

In contrast, one participant (SAW10), despite practicing SMBG twice a week, viewed it as useless, saying that SMBG is “just testing where you are right now. It’s not medicine. It doesn’t motivate you do to anything”. She also viewed SMBG as wasteful and costly, particularly because she believed that she could gauge her blood sugar highs and lows based on bodily cues and symptoms.

### Theme three: perceived barriers of SMBG

All participants identified barriers that may inhibit regular SMBG use. Five were noted most often: negative emotional responses to unexpected blood glucose readings, cost, pricking pain, burden of SMBG, and lack of self-discipline/motivation.

First, some participants experienced emotional distress from SMBG readings. As one participant explained, “So why would I just do it and find out, ‘Oh it’s 8 or 9’, and then get depressed. So I wouldn’t. Why would I do it when I know it’s high?” (SAW10). Although SMBG results encouraged individuals to reflect upon their self-care practices, when the readings caused confusion some participants felt frustrated and disheartened:

**CW1:** Sometimes I feel like giving up, I have to be honest, I feel frustrated or dumb in spirit, you know, at times, because, um, no matter how much I exercise and try to eat right and I take the medication, I get unusual readings sometimes. Because, as I said before, for the first reading of the week it’s good, but after each meal it just escalates. Even if I eat the right, you know, diet or meal – follow the right meal plan, it’s scary sometimes.

Second, the cost of lancets and strips inhibited most participants from regular SMBG. Compared to Caribbean participants, South Asian participants were more vocal about these costs:

**SAW10:** She [her physician] told me that you should monitor it several times before meals, after meals, before sleeping, getting up, to see a pattern. So, like, the strips are very expensive… That I’m wasting this one, this one dollar is gone. How will I be able to buy more strips? Like, the bundle costs $100 for 100 strips. Then I get lots of resistance from my family. My husband will say, ‘Why are you checking it?’ I don’t know if that is the same issue in his mind too. So, well, it’s not a very enjoyable exercise.

A few participants reported that to reduce the cost, they reused lancets which made lancing their skin much more painful:

CW1: Sometimes I run out of the lancets because I have to pay for them at the pharmacy. And, sometimes, I have to use the same needle over and over again. Whenever I use an old needle it pricks me harder, y’know, it goes down deeper in my skin. It really hurts. And I have to use the rubbing alcohol to massage it as firmly as possible. So I try to make the sacrifice. When I have the money, I try to make the sacrifice. Especially this month I am buying two cases of the lancets so that they last for the rest of the month, because sometimes one package just lasts for a week because of the frequency in which I have to do the testing.

Caribbean participants cited pricking pain as a barrier more often than South Asian participants, as they tended to perform SMBG more frequently. For one participant, pricking was the only reason he avoided SMBG some days:

CM5: Because I’m tired of pricking my fingers. Pricking, pricking, pricking. Sometimes I feel it so, so, you know the nerves are right at the tips of your fingers and your toes there, the nerves are right there, and um I could feel it, it hurts, you know. That’s why I think sometimes, you know, I skip couple of days, two, three days.

Fourth, two South Asian women commented on SMBG being a “hassle”, a technical nuisance: “Besides, the machines are broken, machines, batteries they get expired. And you don’t know how to put the batteries back in. You have to go to a [pharmacy]. It is such a hassle” (SAW10). Another woman emphasized the repetitive burden of SMBG, that she feels is forced upon her, although this burden did not impede regular monitoring:

**SAW11**: I mean if it’s high today, say 7, 7.7, 7.8, then I see that I change my diet like that for diabetes, then I test the next day as well. A couple of days in a row. I mean, then it bothers you, I mean, testing in a row. Otherwise it’s OK. And within two or three days it comes back to around 7-ish. The idea of, I mean the hassle of, just taking it out of the drawer – that’s, that’s the hassle. That’s the hassle. When there is something you have to do by force, then it’s a hassle.

Fifth, rather than identifying external sources of barriers, two South Asian women internalized their barriers and blamed themselves for not practicing SMBG regularly. One (SAW7) voiced her perceived carelessness: “No, no, it’s not the right attitude, it’s not the right thing, I know it’s not right. I should be monitoring, I should be taking more care of my food habits. I’m just careless about it, I know I’m careless.” Similarly, SAW10 blamed her lack of routine: “I’m not a very regular and organized person, so I’m not taking advantage of it [SMBG]. I should make a routine. I should try to be on time, check it in the morning and then try to act accordingly”.

### Theme four: cues to action

The cues to action that participants most often described were support from physicians, family members, and friends. First, most participants said that their physicians regularly talked to them about how often to practice SMBG and gave them log books for recording their blood sugar levels. Physicians also adjusted participants’ treatment regime when their readings were too high. In contrast, a few participants who monitored infrequently (SAW7 and SAW10) did not receive consistent cues for action from their physicians, which may have affected these participants’ practice of SMBG. Indeed, ongoing encouragement from physicians may be necessary to motivate SMBG practice. When there are no physician cues to action, patients may perceive that their physicians do not value SMBG and then perform it less often or not at all. One participant began to doubt the value of SMBG when her physician did not reinforce its practice:

**Interviewer:** Does she think it is important for you to test?

**SAW10:** I don’t know. I don’t remember.

**Interviewer:** Do you get a sense from her?

**SAW10:** No, because, you know, when we see each other, it is after many months and then for only 5–6 or 7 minutes. She quickly looks at my previous report and then she says you are doing fine, or you are not doing fine, you should exercise, and that’s it. If I trust the doctor, then I wouldn’t do it [SMBG]. I would trust. He or she knows best, so why should I do it [SMBG]? It’s [SMBG] not a very comfortable thing, so why would I do it?

Second, most participants spoke about the prevalence of diabetes in their families and communities. For most participants, having family and friends with diabetes helped them cope with diabetes management. Those who practiced SMBG, particularly Caribbean participants, either received or sought support from family members. However, South Asian women participants reported that they received limited family support. As one participant explained, her husband objected to her testing and appears to deny that she even has diabetes:

**SAW10:** Like I said… I think I am mentally more busy than I really am. Family, my husband. Family is a demanding situation. It’s a very demanding situation…And, so I am very careful about that. I just do [SMBG] once in the morning sometimes. So that’s one reason. And then I don’t have a support system from my family. So my husband is very, very discouraging, in that he says, ‘You say to yourself you don’t have anything’. But he sees me doing that [SMBG], he gets so upset, he gets really hyper – ‘You don’t have anything’ – and like that [laughs]. So, you know, all these things, they hurt. Like my mom tells me that ‘You should be very careful’ [about diabetes], but I don’t.

### Theme five: self-efficacy

The key findings under this theme pertain to the confidence participants expressed in practicing SMBG and strategies for overcoming barriers. First, all participants said that SMBG was easy to do and all felt confident about their ability to use the glucometer, prick themselves and read the results. For instance, when asked whether anything could stop him from using SMBG, one participant responded, “I don’t think anything can stop me. Even if I travelled I can still test it” (CM8). Another said, “I try to test my blood sugar every morning. Even if I go away for a weekend, I take everything with me so I can test. Because I like to know each morning what the reading is” (CW3).

A final important finding was the various strategies participants used to overcome barriers to SMBG. These strategies, which demonstrated participants’ problem-solving skills, included reusing SMBG materials, such as lancets, for financial reasons, and owning backup glucometers in case one provided questionable results.

## Discussion

Using the HBM, this study explored the views and practices of SMBG among Caribbean and South Asian non-insulin-treated individuals with Type 2 DM. With the exception of one participant, who was newly diagnosed and the least likely to practice SMBG, most participants believed that diabetes is a serious condition, and expressed concern about their susceptibility to developing future complications. Patient beliefs regarding severity and susceptibility are important, as these perceptions influence preventative self-care behaviours [28] and observance of self-management recommendations [31], such as SMBG. Most participants found SMBG useful in their daily self-management routines and were confident in their ability to use the tool. Although all participants practiced SMBG, the frequency varied. Most Caribbean participants practiced SMBG more frequently, once a day or more, compared to once a day or less for most South Asian participants. At the time of this study, guidelines recommended that the frequency of SMBG should be individualized [32], resulting in physicians and diabetes educators using professional discretion in recommending SMBG frequency.

Participants commonly described SMBG as a feedback tool that helps them understand and gauge the impact of other self-management activities, such as diet, physical activity, medications, in controlling blood glucose levels. Although this finding is consistent with the literature [20,33,34], it emphasizes the benefits that SMBG could have in helping people modify their self-care behaviours. Similar to Peel et al. [24,25], some participants described SMBG as an integral part of their diabetes self-management that facilitated control and understanding of their illness. This was particularly true for one asymptomatic participant, as testing showed him where he “stands” physiologically. SMBG’s importance was captured in phrases such as “lifeline”, “it’s like medication”, and as being their “duty” and “own doctor”, demonstrating participants’ sense of responsibility in managing the condition.

The most common barrier reported by the participants was SMBG’s expense. This finding is well supported in the literature [34-36], and it is likely that SMBG practice would increase if related expenses were minimized or eliminated [18,36]. In Ontario, the average cost per SMBG strip is 72 cents, while in the rest of Canada, it is almost a dollar [16]. Most of the participants in our study were under the age of 65 and therefore not covered by the Ontario Drug Plan, which reimburses SMBG expenses. There is mounting evidence that SMBG is not cost-effective and many advocate that the frequency of SMBG for patients with Type 2 DM not treated with insulin should be limited (i.e., 1–2 times per week) [12,37], which can incur a substantial reduction in expenditures [17]. In response to this evidence, the Canadian Diabetes Association released more specific SMBG guidelines stating that patients who are not treated with insulin, have stable and controlled blood glucose levels, and are not at risk of hypoglycemia would benefit from performing SMBG less than 2 times per week [38]. However, they recommend a minimum government reimbursement of 30 strips per month for this group (approximately 7-8 strips per week; around 1 test per day) [1]. Given these recent recommendations on SMBG, it is important that healthcare professionals assess patients’ views, preferences and unique circumstances before recommending whether and how often SMBG should be used. This is particularly important because, as our findings revealed, some participants tried to reduce the expense of SMBG by reusing lancets, which is not a recommended practice as it can cause more pain when pricking [39].

Our finding that SMBG can trigger both positive and negative emotional responses, depending on the reading, corresponds with the literature that suggests that there is a relationship between distress and SMBG practice among non-insulin treated individuals with diabetes [20,21]. Readings within recommended targets encouraged participants such that they felt “happy” that they are managing well. In contrast, when readings were outside the target range, participants feel disappointed, frustrated, anxious and sad. These responses are consistent with Peel et al. [20] who found that newly diagnosed patients feel gratified when readings were low and feel like “failures” when readings were too high. Because SMBG may produce a sense of disappointment when readings remain high, which can result in emotional distress, health care providers must educate patients on the reasons why SMBG readings may be inexplicable and contradictory; for example, weight gain, new medications or increase in medication dose, gastroparesis, illness or infection, may all influence blood sugar levels.

We observed two South Asian women (SAW7 and SAW10) blaming themselves for lacking the self-discipline to practice SMBG and other diabetes self-management activities, which was also found among women participants in Peel et al. [20,21]. Research has shown that negative coping strategies, such as self-blame, in diabetes management occur more often among women than men [40], which can potentially be explained by the lack of social support that women receive. In a meta-synthesis, Gomersall et al. [41] report that women tend to construe diabetes self-management as their own responsibility that needs to be negotiated with other family members, and that the needs of spouses and children typically take precedence over women’s needs. We noted this contextual issue in our study where two South Asian women expressed the demands of family life and lack of family support in relation to SMBG. These findings demonstrate that diabetes self-management is influenced by multiple contexts and sociocultural factors that are not directly under the control of the individual, which may undermine personal agency to maintain self-management [40]. It is important that health professionals are aware of the gendered dimensions of diabetes self-management and how that may differ across cultures.

The literature suggests that physicians play a crucial role in patients’ use of SMBG [13,20,21,42]. Although most of the participants strongly believed in the importance of SMBG as part of diabetes management, they acknowledged that physicians’ regular encouragement reinforced their practice. Yet, one participant whose physician did not consistently remind her to monitor, did not view SMBG as a useful tool for diabetes management. This finding is consistent with Peel et al.’s [20,21] findings that patients discontinue using SMBG and view it as a waste of time if they sense that their physicians do not value SMBG or do not use its results to make therapeutic decisions. A Canadian study found that healthcare providers generally value SMBG as it allows them to identify blood-glucose patterns that present educational opportunities for diabetes self-management [41]. This study also concurs with our findings that, if healthcare professionals view SMBG as beneficial and use its results to engage patients in dialogue, then patients are likely to perform SMBG [42], highlighting the influence health care providers have in patient views and practice of SMBG.

This study has limitations that must be noted. First, our sample size of 12 may be considered a limitation as it may not have allowed for data saturation. However, many of our findings support the existing literature on patients’ views on SMBG, which has been conducted with white participants, and show similar themes emerging from the two cultural populations represented in this study. Nevertheless, discussions that are based on two or less participant quotes are too preliminary to draw strong conclusions. Another limitation is our recruiting method, as we recruited participants from two different cultural groups from different diabetes education programs and health centres. These centres could have different quality levels of patient care and services, which in turn could have influenced study participants’ views and practice of SMBG, and hence a potential selection bias based on centre recruitment. Also, the fact that most participants describe SMBG as a valuable self-management tool can potentially be due do wanting to respond in a socially desirable manner. Finally, we did not use ethnically matched interviewers for our study participants, which could have facilitated in generating more culturally specific knowledge.

## Conclusion

Individuals with diabetes are major stakeholders in the management of their condition and they should therefore be involved in all decisions regarding their care. This study is the first to specifically address the perspectives of ethnic minority patients’ about SMBG. Overall, our findings show similar themes emerging from Black Caribbean and South Asian populations compared to the sparse literature on patients’ views on SMBG, which predominately focused on white participants. However, differences in self-blame and social support with regards to the practice of SMBG were observed across gender and culture that should be noted to better support diabetes self-management. Using SMBG can empower patients to better understand and maintain their diabetes self-care behaviours. Healthcare professionals play a key role in the way SMBG is perceived and used by patients not on insulin. Given the financial and emotional implications of practicing SMBG, it is important for healthcare providers to consider patients’ clinical, financial and social context, prior to advising the use and frequency of SMBG among non-insulin treated individuals with diabetes. Further research is needed to corroborate our findings and to give voice to ethnic populations with a disproportionate burden of diabetes on their perspective of self-management issues.

## Competing interest

The authors declare that they have no competing interests.

## Authors’ contributions

Study design and concept: EG, HB, EM and EP. Analyses of data: EG, MF, and AS. Interpretation of results: EG, MF, AS, HB, EM and EP. Drafting of the manuscript: EG, MF, AS, HB, EM, and EP. All authors read and approved the final manuscript.

## Pre-publication history

The pre-publication history for this paper can be accessed here:

http://www.biomedcentral.com/1472-6823/13/46/prepub
